# Concomitant Removal of Perfluorooctanoic Acid (PFOA) and Humic Acid from Water Using a Cationic Cellulose Nanofiber

**DOI:** 10.1021/acsomega.6c02078

**Published:** 2026-05-19

**Authors:** Azinsadat Jahani Javanmardi, Merle Plassmann, Aji P. Mathew

**Affiliations:** † Department of Chemistry, 7675 Stockholm University, Stockholm 106 91, Sweden; ‡ Department of Environmental Science, Stockholm University, Stockholm 106 91, Sweden

## Abstract

Per- and polyfluoroalkyl substances (PFAS), particularly perfluorooctanoic acid (PFOA), are persistent, mobile, and difficult-to-remove contaminants in water systems. This study investigates a hybrid adsorption system combining cationic cellulose nanofibers (Cat-CNF) with humic acid (HA) in solution to enhance the removal of PFOA from high-concentration contaminated water sources, such as AFFF-impacted groundwater, industrial effluents, and landfill leachates. Batch experiments spanning a wide concentration range (200–600,000 μg L^–1^ PFOA), pH (4.0–11.0), and HA loading (20–100 mg L^–1^) revealed that HA strongly binds to Cat-CNF, forming hydrophobic domains that promote PFOA uptake via combined electrostatic and hydrophobic interactions. PFOA adsorption remained high across the full pH range, especially at elevated HA concentrations, and was unaffected by competitive HA presence. Kinetic studies showed that over 90% of PFOA uptake occurred within the first 90 min, reaching equilibrium within 4 h. Isotherm studies showed a steep uptake curve and saturation at ∼250 mg g^–1^, with strong fits to both Langmuir (*R* ^2^ = 0.98) and Freundlich (*R* ^2^ = 0.89) models. Regeneration experiments demonstrated stable HA retention and progressive improvement in PFOA desorption over multiple cycles, confirming the system’s reusability. These findings highlight the Cat-CNF/HA hybrid as a robust and regenerable adsorbent with rapid equilibrium attainment (within 4 h) for PFOA remediation in high-contamination scenarios, with HA contributing both to contaminant binding and adsorbent performance.

## Graphical abstract

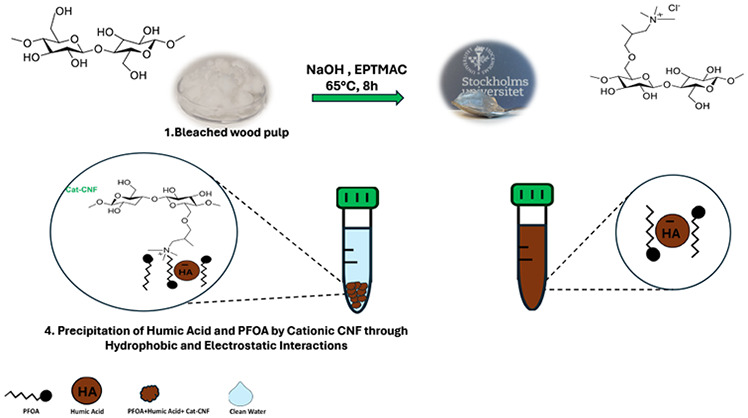

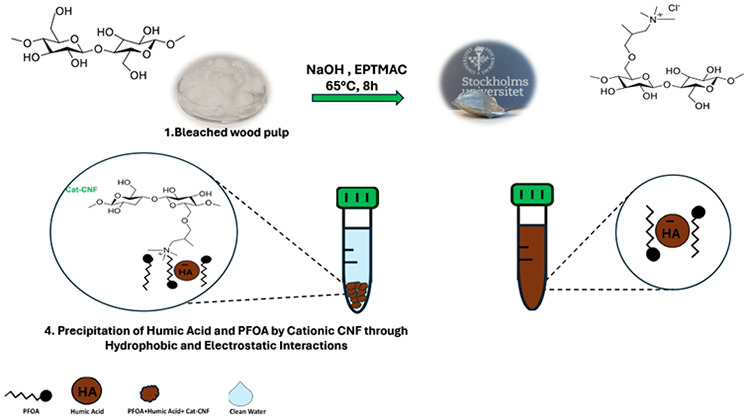

## 1 Introduction

Perfluoroalkyl and polyfluoroalkyl substances (PFASs) are synthetic fluorinated organic compounds characterized by strong C–F bonds that confer exceptional chemical and thermal stability.^1,2^ PFAS contamination is widespread in Europe and the United States, mainly from industrial sources, airports, and firefighting foams. In the Czech Republic, PFAS were found in over 70% of drinking water samples, while levels in southern Lyon, France, reached 695 ng/L near fluoropolymer plants. The EU Drinking Water Directive (2020/2184) sets limits of 100 ng/L for 20 PFAS and 500 ng/L for total PFAS from 2026, with Denmark and Sweden already enforcing stricter thresholds of 2–4 ng/L. In 2024, the U.S. EPA introduced Maximum Contaminant Levels of 4 ng/L for PFOA and PFOS, and 10 ng/L for PFHxS, PFNA, and GenX. These measures reflect growing recognition that PFAS pose health risks even at trace levels.^3–6^ Beyond diffuse environmental contamination, point-source scenarios such as aqueous film-forming foam (AFFF)-impacted groundwater at military and commercial airports, industrial effluents, and landfill leachates can exhibit PFOA concentrations orders of magnitude higher, reaching up to 4,470 ng/L at firefighting training sites in Sweden and up to 6.6 mg/L in heavily contaminated groundwater globally.^7,8^ Effective remediation of these high-concentration sources requires adsorbent systems capable of performing across a wide concentration range.

Several technologies have been investigated for PFAS removal, including advanced oxidation, ion exchange, membrane separation, and adsorption. Of these, adsorption with granular or powdered activated carbon (GAC, PAC) remains the most widely applied. However, carbon adsorbents are limited by fossil-based sourcing, energy-intensive regeneration. Their negatively charged surfaces also hinder electrostatic interactions with anionic PFAS, resulting in incomplete removal. While uptake is largely governed by hydrophobic interactions that increase with chain length, electrostatics may contribute when surface modification is applied. Alternative technologies, including ion exchange resins, fluorinated adsorbents, and membrane processes, often concentrate PFAS into secondary waste streams that require additional treatment.^7,9–13^

Among PFASs, perfluorooctanoic acid (PFOA) is among the most widely detected in the environment, raising significant concern due to their persistence, bioaccumulation potential, and adverse effects on human health and ecosystems.^14^ Studies indicate that the adsorption of PFOA is largely governed by a balance of electrostatic forces, due to its negatively charged headgroup, and hydrophobic interactions arising from its perfluorinated carbon chain.^15,16^ The presence of dissolved organic matter (DOM) significantly affects PFAS adsorption by altering electrostatic and hydrophobic interactions. Negatively charged DOM can introduce electrostatic repulsion on adsorbent surfaces, thereby reducing PFAS uptake, while its aromatic and hydrophobic domains may facilitate adsorption through coassociation with fluorocarbon chains. In addition, DOM can induce fouling via surface complexation or coagulation, particularly in systems where leached metal ions interact with humic substances. Despite these influences, the role of DOM is often underestimated, as most adsorption studies evaluate PFAS removal in the absence of organic matter.^17^

Humic acid (HA), a major fraction of dissolved organic matter (DOM),^18^ is an amphiphilic macromolecule characterized by heterogeneous structures and abundant functional groups. Its pH-dependent solubility and tendency to self-assemble into supramolecular aggregates enable interactions with both hydrophilic and hydrophobic substances. In water treatment, HA functions effectively as a natural biocoagulant and bioflocculant, promoting the removal of suspended particles, colloids, metals, and organic pollutants through adsorption, complexation, and precipitation. These properties not only improve water clarity and reduce turbidity but also allow HA to act as a carrier for hydrophobic pollutants, thereby influencing their mobility and potential biodegradation.^19,20^ Although humic acid exhibits promising adsorption and flocculation properties in water treatment, its potential as a functional coadsorbent in hybrid systems remains largely unexplored. Most existing studies treat HA as an interfering cocontaminant rather than a performance-enhancing component, overlooking its amphiphilic nature and ability to introduce hydrophobic domains that could facilitate PFAS uptake alongside electrostatic interactions.

Cellulose-derived materials are especially promising due to their abundance, tunable chemistry, and functionalization potential.^21^ Among them, cationic cellulose nanofibrils (Cat-CNF) provide high surface area, stable positive charge, and sustainable sourcing, positioning them as strong candidates for efficient PFAS remediation.^22–25^

Herein, we developed a hybrid adsorption system by introducing humic acid (HA) as a coadsorbent in solution and quaternized cellulose nanofibrils (Cat-CNF) as the primary adsorbent to enhance the removal of perfluorooctanoic acid (PFOA) from water. Cat-CNF was synthesized by modifying cellulose with glycidyltrimethylammonium chloride to introduce quaternary ammonium groups, followed by mechanical fibrillation. The material was characterized using scanning electron microscopy (SEM), atomic force microscopy (AFM), energy-dispersive X-ray spectroscopy (EDS), and zeta potential measurements to confirm successful surface modification and adsorbate interactions. Batch experiments were conducted to investigate the effects of pH, HA concentration, and PFOA loading on adsorption behavior. Additionally, kinetic, isotherm, and regeneration studies were performed to evaluate the system’s removal performance and reusability. Quantitative analysis of PFOA and HA concentrations before and after treatment was conducted using ultrahigh-performance liquid chromatography coupled with tandem mass spectrometry (UHPLC–MS/MS) and UV–Vis spectrophotometry, respectively.

## 2 Materials and Methods

### 2.1 Chemicals and Materials

(2,3-Epoxypropyl)­trimethylammonium chloride (EPTMAC, ∼80% in water, TCI Chemicals), 5 μm nylon mesh (Sefar Nitex 03-5/1), hydrochloric acid (HCl, 37 wt %, AnalaR Normapur), humic acid (Carl Roth, virgin, laboratory grade), methanol (Optima grade, VWR), perfluorooctanoic acid (PFOA, Supelco, VWR, analytical standard), bleached wood pulp (FCBA), RC syringe filters (0.2 μm, 26 mm) and syringe (Phenomenex), silver nitrate (ACS ≥ 99%, Sigma-Aldrich), sodium hydroxide pellets (NaOH, >98%, Honeywell Fluka), and standardized sodium hydroxide solutions (NaOH, 0.01 and 0.1 M, Fisher Chemical).

### 2.2 Preparation of Cationic Cellulose Nanofiber (Cat-CNF)

Poplar (Kostar) wood was subjected to alkaline pulping using a 10 wt % KOH solution at a solid-to-liquid ratio of 1:20. The treatment was conducted at 80 °C for 4 h under continuous stirring. After pulping, the resulting material was washed thoroughly with deionized water to remove residual alkali. The pulp suspension was subsequently neutralized to pH 6.5–7.5 using HCl and further washed with deionized water until the conductivity decreased to 10–20 μS cm^–1^. The obtained pulp was stored at 4 °C prior to further use. For the synthesis of cationic cellulose nanofibers (Cat-CNF) form the pulp a slightly modified from previously reported procedures was used.^26^ Briefly, bleached pulp fibers were dispersed in an aqueous NaOH solution (5 wt % NaOH, 2.5 wt % fibers) and stirred overnight at 65 °C to enhance the reactivity of hydroxyl groups of cellulose. Subsequently, glycidyltrimethylammonium chloride (EPTMAC) was introduced at a molar ratio of 1:10 (hydroxyl groups to ammonium groups) to initiate cationization. The reaction proceeded under continuous stirring for 8 h at 65 °C. Upon completion, the suspension was acidified by dropwise addition of HCl to neutralize the residual base. The reaction mixture was then rinsed with anhydrous ethanol to remove unreacted EPTMAC. The modified fibers were collected by vacuum filtration using a 5 μm nylon mesh and thoroughly washed with deionized water until the conductivity of the filtrate dropped below 10 μS cm^–1^. The resulting pulp was diluted to ∼0.3 wt % and passed five times through a high-pressure microfluidizer (LM10 Microfluidizer) equipped with a 100 μm interaction chamber at 1500 bar to obtain the Cat-CNF.

### 2.3 Cat-CNF Characterization

Cationic cellulose nanofibers (Cat-CNF) were characterized using spectroscopic and microscopic techniques. FTIR spectra were collected on a Varian 670-IR spectrometer equipped with an ATR accessory using dried samples of Cat-CNF and bleached pulp. Measurements were conducted over the range of 500–4000 cm^–1^ with a resolution of 4 cm^–1^ and averaged over at least 32 scans.^27^ Surface charge and degree of substitution (DS) were determined by conductometric titration using 0.1 M AgNO_3_.^28^ Surface charge behavior as a function of pH was assessed by ζ-potential measurements^29^ using a Zetasizer Nano ZS (Malvern Instruments, UK). Measurements were conducted at 25 °C over a pH range of (2–12). Nanofiber morphology was characterized by AFM^30^ and cryo-SEM.^31^ For AFM imaging, Cat-CNF was diluted to 0.005 wt %, sonicated, and drop-cast onto mica, followed by air drying. Images were collected in peak-force tapping mode using a Bruker Multimode-8 instrument. Cryo-SEM was employed to visualize gel-phase Cat-CNF samples before and after adsorption of contaminants. Samples were plunge-frozen in liquid nitrogen (−196 °C), transferred under cryo-vacuum, and coated with a 4 nm gold layer. Imaging was carried out using a JSM-IT800 microscope under cryogenic conditions to preserve fibril structure and capture morphological changes associated with humic acid and PFAS interaction. Full experimental details are provided in the Supporting Information.

### 2.4 Water Sample Preparation

#### 2.4.1 Humic Acid Stock

Commercial humic acid was purified prior to use. HA was dissolved in 0.1 M NaOH and filtered through a 0.45 μm membrane. The resulting solution was acidified to pH 1.0 using 1 M HCl to induce precipitation. Precipitates were separated by centrifugation, rinsed thoroughly with 0.1 M HCl and deionized water, and freeze-dried. A 100 mg L^–1^ stock solution of the purified HA was prepared in deionized water and adjusted to pH 7.0.^32^

#### 2.4.2 PFOA Stock

Aqueous stock solutions of PFOA were prepared in deionized water at target concentrations of (200–600,000 μg L^–1^). Working solutions were freshly prepared for each experiment, and pH adjustments (pH 4.0, 6.5, and 10.5) were made using 0.1 M HCl or 0.1 M NaOH. All solutions were handled in polypropylene containers to minimize sorption losses.^22^

### 2.5 Water Analysis

#### 2.5.1 Humic Acid Analysis

HA concentrations were determined using a Cary 5000 UV–Vis spectrophotometer^33^ (Agilent Technologies) at 254 nm in a 1 cm quartz cuvette. Separate calibration curves were constructed for each pH condition due to the pH dependence of HA absorbance.

#### 2.5.2 PFAS Analysis

PFOA concentrations were quantified using ultrahigh-performance liquid chromatography coupled with tandem mass spectrometry^34,35^ operated in negative electrospray ionization (ESI^–^) mode. Diluted filtrate samples were analyzed using chromatographic and MS conditions as detailed in the Supporting Information.

### 2.6 Adsorption Studies

#### 2.6.1 Isotherm Studies

Isotherm experiments were carried out at three initial concentrations: (20–300 mg L^–1^) for HA, and (200–600,000 μg L^–1^) for PFOA. Adsorption was performed in 20 mL polypropylene centrifuge tubes at a fixed Cat-CNF concentration of 1 g L^–1^. The pH of the working solutions was adjusted to 4.0, 6.5, or 10.5 using 0.1 M NaOH or HCl. Tubes were shaken at 150 rpm and 25 °C for 72 h to ensure equilibrium. After centrifugation (4000 rpm, 10 min), supernatants were collected for analysis. For PFOA, samples were further filtered through 0.2 μm RC syringe filters before quantification. All conditions were tested in duplicate.^22^

#### 2.6.2 Kinetic Studies

Kinetic experiments were conducted to evaluate the adsorption behavior of Cat-CNF toward PFOA and humic acid (HA). PFOA and HA were prepared at concentrations of 2000 μg L^–1^ and 60 mg L^–1^, respectively, with Cat-CNF fixed at 1 g L^–1^. Samples were agitated on an orbital shaker at 150 rpm and 25 ± 1 °C. At selected time intervals, aliquots were collected, centrifuged at 4000 rpm for 10 min, and filtered through 0.2 μm regenerated cellulose (RC) syringe filters. Control experiments confirmed that no measurable PFAS was retained on the filter. Supernatants were analyzed for residual HA and PFOA concentrations. All experiments were conducted in duplicate.

#### 2.6.3 Regeneration Experiments

To assess reusability, Cat-CNF samples loaded with HA or PFOA were subjected to three consecutive adsorption–desorption cycles. Desorption was conducted by treating the adsorbent with 0.6 M NaCl at 150 rpm and 25 °C for 24 h. Following centrifugation, the supernatant was collected and the Cat-CNF was rinsed with deionized water to remove residual salt. The regenerated adsorbent was then reused for a new adsorption cycle under identical conditions. Performance was evaluated by comparing removal efficiencies across cycles. Each cycle was conducted in duplicate.

## 3 Results and Discussion

### 3.1 Cat-CNF Characterization

Quaternization was employed to introduce cationic functionality onto cellulose nanofibrils (CNF), using a mild, aqueous-phase reaction designed to align with green chemistry principles. Sodium hydroxide (5 wt %) was used to activate hydroxyl groups on the cellulose surface, facilitating nucleophilic attack by (2,3-epoxypropyl)­trimethylammonium chloride (EPTMAC). A molar ratio of 10:1 (EPTMAC:hydroxyl groups) was selected to promote efficient surface functionalization while minimizing structural degradation. Excessive NaOH or EPTMAC may induce structural degradation or reduce crystallinity of cellulose.

Figure 1a shows FTIR spectra of bleached pulp and Cat-CNF, confirming the presence of functional groups associated with quaternary ammonium moieties. Both unmodified pulp and Cat-CNF exhibited characteristic cellulose peaks in the 500–1400 cm^–1^ range, including a C–O–C pyranose band near 1050 cm^–1^ and a broad −OH stretch between 3200–3500 cm^–1^. A distinct peak at 1480 cm^–1^ was observed exclusively in the Cat-CNF sample, attributed to methyl vibrations in the trimethylammonium groups. A shoulder near 1640 cm^–1^, present in both spectra, may be associated with bound water or quaternary nitrogen vibrations.

**Figure 1:**
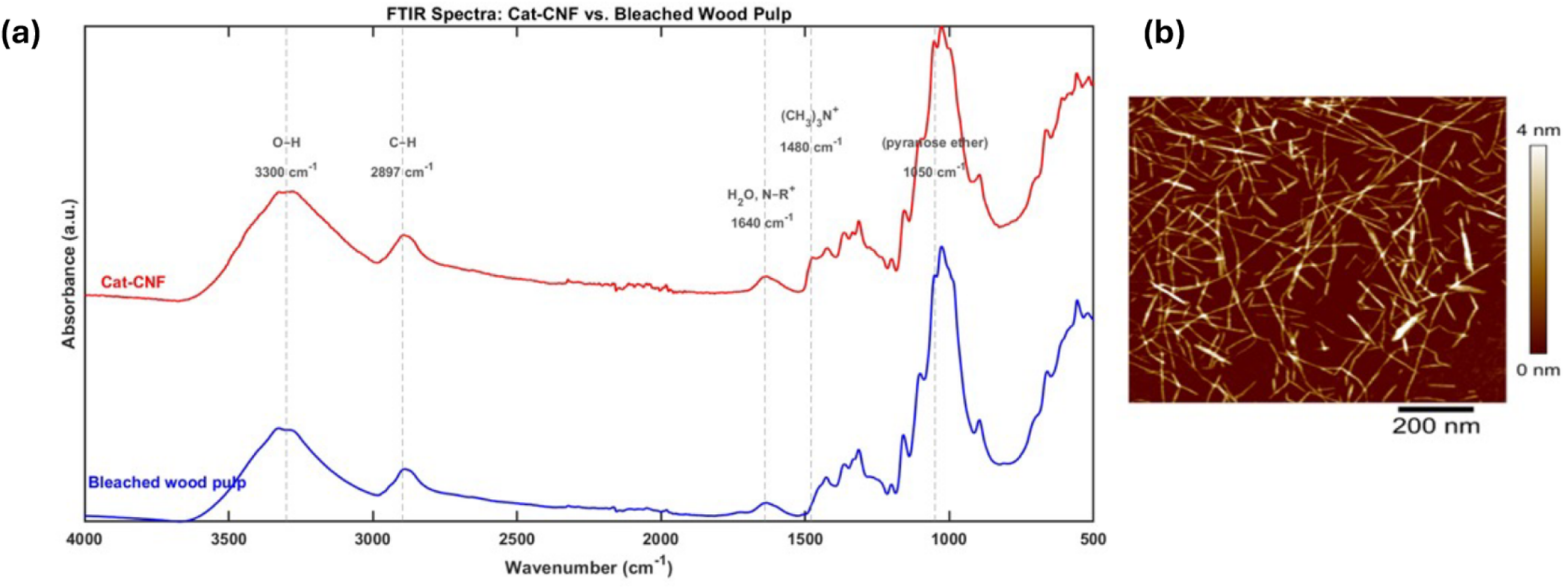
(a) FTIR spectra of bleached wood pulp and cationic cellulose nanofibers (Cat-CNF). (b) Atomic force microscopy (AFM) image of (Cat-CNF).

AFM imaging of Cat-CNF revealed a well-dispersed nanofibril network with high aspect ratio and limited aggregation (Figure 1b). The average fiber width was estimated at 4 ± 1.35 nm. When diluted to 0.005 wt %, the dispersion exhibited high transparency and viscosity, consistent with effective fibrillation and formation of a gel-like nanofiber matrix.

Surface charge measurements by conductometric titration indicated a quaternary ammonium content of 0.75 mmol g^–1^, corresponding to a degree of substitution (DS) of approximately 0.14. Zeta potential measurements confirmed the stability of the cationic surface charge over a pH range of 2–10, indicating covalent attachment of the functional groups and resistance to dissociation.

### 3.2 Adsorption of Humic Acid on Cat-CNF

#### 3.2.1 Effect of pH and Concentration

The influence of initial humic acid (HA) concentration and pH was examined to assess the adsorption performance of Cat-CNF under environmentally relevant conditions. As shown in Figure 2, both the adsorption capacity (Q_e_) increased with increasing HA concentration (20–300 mg/L) at all pH levels, indicating that Cat-CNF provided abundant active sites and was not saturated within this range. This concentration-dependent behavior is consistent with the results reported by Sehaqui et al.^36^ confirming that adsorption is primarily governed by electrostatic attraction between the anionic functional groups of HA and the cationic quaternary ammonium sites on Cat-CNF.

**Figure 2:**
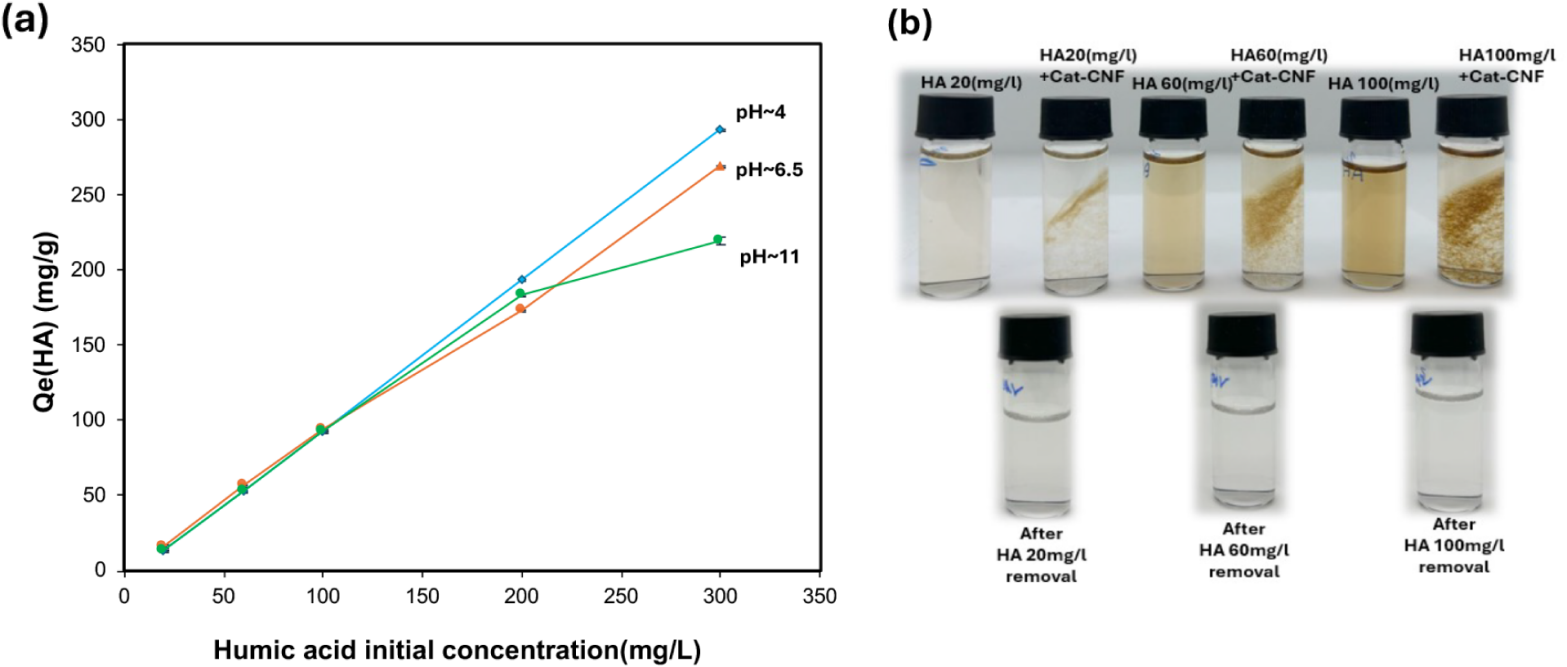
(a) Effect of pH on humic acid adsorption capacity (Q_e_) by Cat-CNF at different initial HA concentrations (b) Photographic images of HA solutions before and after treatment, showing HA alone, HA mixed with Cat-CNF, and the corresponding supernatants after removal at 20, 60, and 100 mg/L initial concentrations at pH = 4.0 ± 0.2.

Literature shows that HA forms compact aggregates at low pH and extended coil-like conformations at high pH due to the ionization of its carboxylic and phenolic groups.^37–41^ In this study although HA exhibits strong concentration dependent behavior, these pH dependent structural changes appear to have limited effect on overall adsorption at low concentrations. At low and moderate HA concentrations (≤100 mg/L), Q_e_ values remained consistent across all tested pH levels, suggesting that pH had minimal influence on adsorption under dilute conditions.

At higher HA levels (≥200 mg/L), however, the effect of pH became significant (*p* < 0.05). Adsorption decreased under alkaline conditions, from approximately 293 mg/g at pH 4 to 219 mg/g at pH 11. This reduction can be attributed to increased deprotonation and molecular expansion of HA, which reduce electrostatic attraction and partially shield the active sites of Cat-CNF. The zeta potential measurements (S3) confirmed this interpretation, indicating that Cat-CNF retained a strong positive surface charge in the presence of humic acid across a wide pH range, decreasing from +41 mV at pH 4 to +15 mV at pH 11, providing direct evidence of HA binding to the Cat-CNF surface and partial charge neutralization under alkaline conditions. This persistent cationic character maintained favorable electrostatic interactions with anionic HA species even under alkaline conditions. Despite minor reductions in capacity at high concentrations, the removal efficiency remained above 85% for all conditions, demonstrating the robustness of Cat-CNF as an efficient adsorbent for HA across a wide range of pH and concentration levels.

These findings confirm that Cat-CNF provides a highly stable and positively charged surface capable of efficiently binding anionic organic matter such as humic acid across a wide pH range. Given its strong affinity toward HA, Cat-CNF offers an ideal platform for designing hybrid systems where humic acid can act synergistically with perfluoroalkyl substances (PFAS). Therefore, the following section examines the adsorption behavior of PFOA in the presence of HA to evaluate their combined potential for PFAS removal from water.

### 3.3 Interaction between PFOA and HA during Adsorption on Cat-CNF

#### 3.3.1 Influence of PFOA Concentration on HA Adsorption

The effect of PFOA concentration on humic acid (HA) adsorption by Cat-CNF was investigated by varying PFOA levels from 0 to 600,000 μg/L while maintaining HA at 60 mg/L and pH 4. As shown in Figure 3a, the adsorption capacity (Q_e_) of HA remained nearly constant (≈52 mg/g) across all PFOA concentrations, indicating that the presence of PFOA had no measurable influence on HA removal. The Q_e_ values remained highly consistent across all tested PFOA concentrations, ranging from 50.95 ± 0.08 to 52.89 ± 0.35 mg/g, demonstrating that HA uptake by Cat-CNF is unaffected by increasing PFOA concentrations. This behavior can be attributed to the distinct molecular properties of the two adsorbates. HA, a high-molecular-weight polyanionic macromolecule, establishes multiple electrostatic and hydrogen-bonding interactions with the cationic surface, resulting in strong anchoring and limited competition from PFOA. In contrast, PFOA is a small monovalent anion with limited binding multiplicity, which restricts its ability to displace adsorbed HA molecules.^42,43^ Consequently, Cat-CNF exhibits strong selectivity toward HA, maintaining stable adsorption even at elevated PFOA concentrations.

**Figure 3:**
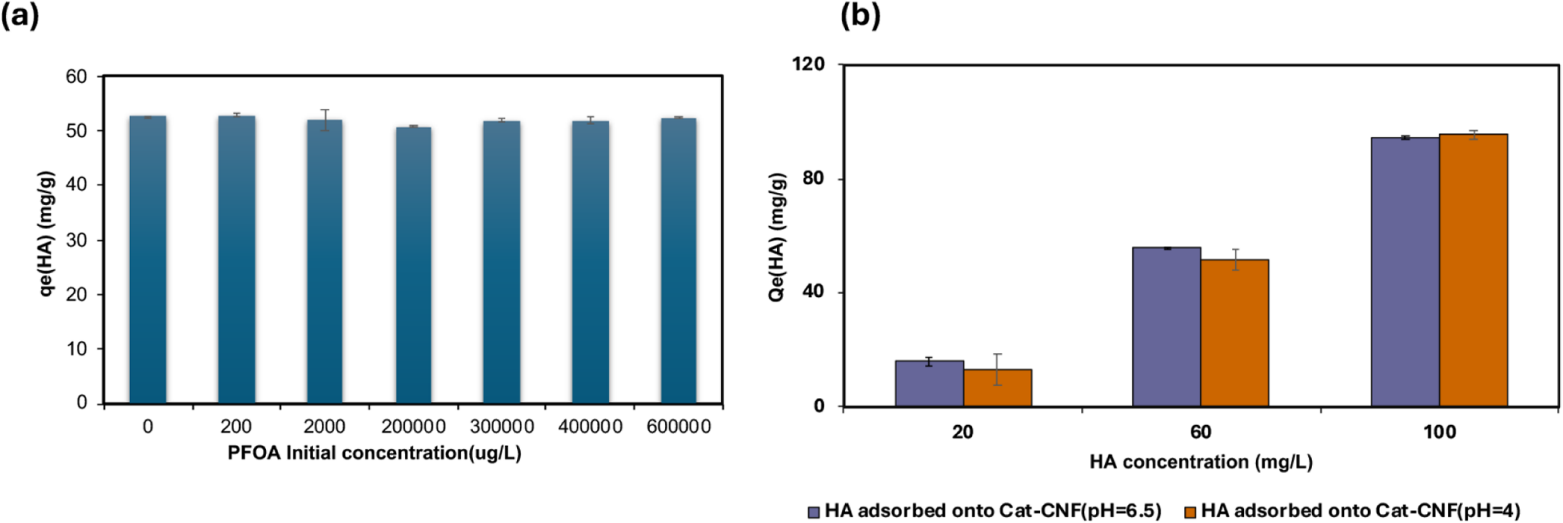
(a) Effect of initial PFOA concentration (200–600000 μg/L) on HA (60 mg/L) sorption by (Cat-CNF). (b) Adsorption of humic acid (20, 60, and 100 mg/L) by Cat-CNF in the presence of PFOA (2000 μg/L) at two pH values (4.0 and 6.5).

A comparable adsorption pattern was obtained when the initial HA concentration was varied (20, 60, and 100 mg L^–1^) at pH 4.0 and 6.5 in the presence of 2000 μg L^–1^ PFOA (Figure 3b). The adsorption capacity increased proportionally with HA concentration, reaching 95.41 ± 4.59 mg g^–1^ at 100 mg L^–1^ HA. The Q_e_ values were comparable between pH 4.0 and 6.5 across all tested HA concentrations, suggesting that pH had minimal influence on HA adsorption in the presence of PFOA. These results confirm that the presence of PFOA does not interfere with HA adsorption by Cat-CNF, demonstrating the stability and surface selectivity of the cationic nanofibers. Building on this stability, the following section investigates the performance of the HA + Cat-CNF hybrid system for the removal of PFOA from water.

#### 3.3.2 Effect of HA on PFOA Adsorption by Cat-CNF

The adsorption performance of Cat-CNF toward PFOA was evaluated at pH 4.0 using three initial concentrations (200, 2000, and 200,000 μg/L) in the absence and presence of 60 mg/L humic acid (HA) (Figure 4a). At the two lower concentrations, both systems exhibited high removal efficiencies, with Cat-CNF alone removing 95.43 ± 1.58 to 97.34 ± 1.52% of PFOA and the Cat-CNF + HA system achieving 97.00 ± 1.00 to 99.79 ± 0.14%. Both systems performed similarly at lower PFOA concentrations, suggesting that HA does not hinder PFOA uptake under dilute conditions, but rather opens up a possibility for concomitant removal of HA and PFOA from aqueous medium.

**Figure 4:**
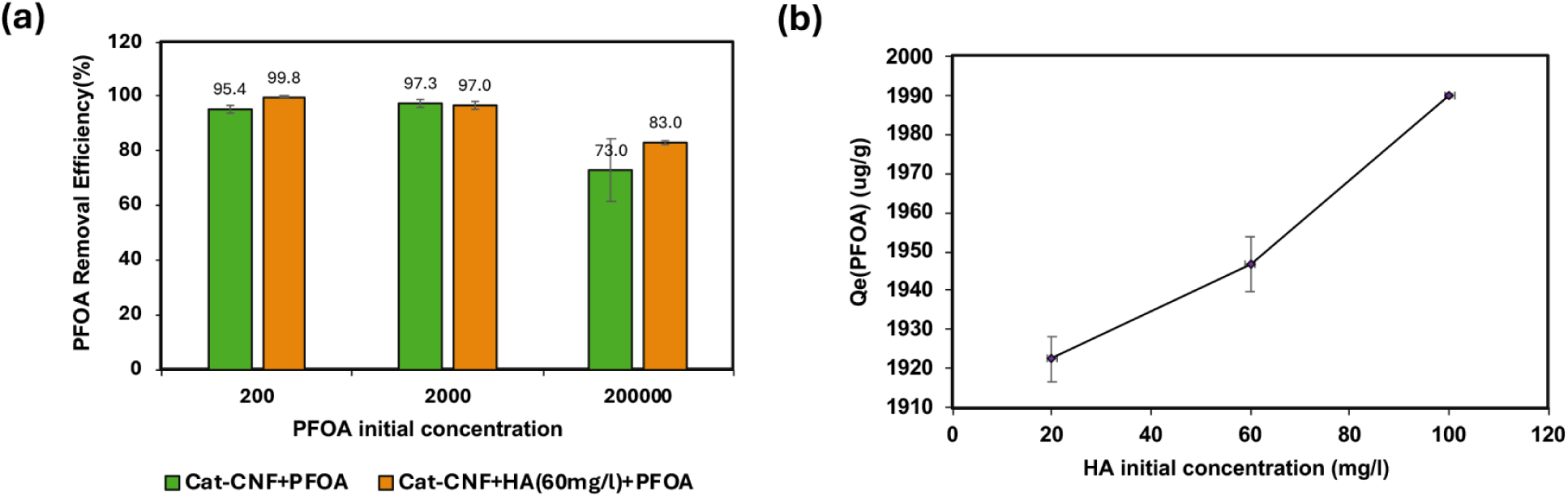
(a) Removal efficiency (RE) of PFOA by (Cat-CNF) at pH 4 across different initial concentrations of PFOA (200, 2000, and 200,000 μg/L). (b) Influence of HA initial concentration on the sorption levels of PFOA on the Cat-CNF (Test conditions:Dry content concentration of Cat-CNF in the solution is 1 g/L, 2,000 μg L PFOA concentration, and pH = 4.0 ± 0.2).

At the highest PFOA concentration (200,000 μg/L), the removal efficiency decreased to 73.03 ± 11.37% for Cat-CNF and 83.00 ± 0.48% for the HA-containing system. The removal efficiency showed a clear concentration-dependent decline, decreasing from 99.79 ± 0.14% at 200 μg/L to 73.03 ± 11.37% at 200,000 μg/L for Cat-CNF alone, and from 97.00 ± 1.00% to 83.00 ± 0.48% for the Cat-CNF + HA system. At lower concentrations, adsorption is primarily governed by electrostatic attraction between the anionic carboxylate headgroup of PFOA and the cationic quaternary ammonium sites on the Cat-CNF surface. As concentration increases, the number of available binding sites becomes limited, resulting in partial saturation and reduced performance.^22^ The slightly higher removal in the presence of HA suggests that HA facilitates PFOA adsorption through hydrophobic association and dispersive interactions. At the same time, the results indicate that electrostatic repulsion between the two anionic species (HA) and (PFOA) does not significantly hinder adsorption under the studied conditions. The decline in performance at high PFOA concentration is therefore attributed to site saturation rather than competitive inhibition by HA, confirming that hydrophobic association remains the dominant mechanism driving PFOA uptake by Cat-CNF.^44–47^ To gain deeper insight into the hydrophobic interactions responsible for the enhanced adsorption, the effect of HA concentration was further investigated by varying HA levels (20, 60, and 100 mg L^–1^) while maintaining a constant PFOA concentration of 2000 μg L^–1^. As illustrated in Figure 4b, increasing HA concentration led to a steady improvement in PFOA uptake. The average adsorption capacity rose from 1922.6 μg g^–1^ (∼96.5% removal) at 20 mg L^–1^ HA to 1989.9 μg g^–1^ (∼99.5%) at 100 mg L^–1^. Correspondingly, the equilibrium PFOA concentration (C_e_) decreased from 77.4 μg L^–1^ to 10.1 μg L^–1^. This progressive improvement with increasing HA loading indicates that higher HA concentrations promote stronger hydrophobic associations and enhance overall PFOA adsorption efficiency. These findings support the interpretation that humic acid enhances PFOA adsorption through cooperative hydrophobic interactions. As HA concentration increases, micelle-like or pseudomicellar aggregates can form, facilitating contact between the nonpolar domains of HA and the perfluorinated chains of PFOA.^48^ As an amphiphilic polyelectrolyte, HA contains both hydrophobic domains and negatively charged functional groups, enabling hydrophobic association with the perfluorinated tails of PFOA and formation of PFOA–HA complexes.^47,49,50^ These complexes enhance PFOA transport and attachment to the positively charged Cat-CNF surface, consistent with previous reports showing that dissolved organic matter can increase PFAS sorption by promoting their dispersion and mobility.^42,46,51^

### 3.4 Effect of pH on PFOA Removal in the Presence of HA

The effect of initial humic acid (HA) concentration on PFOA adsorption by Cat-CNF was evaluated at pH 4.0 and 6.5. As shown in Figure 5, PFOA adsorption was higher at pH 4.0 compared to pH 6.5 when the HA concentration was low (20 mg/L). This enhanced removal can be attributed to the increased protonation of carboxylic and phenolic groups on HA under acidic conditions, which favors stronger electrostatic attraction between the positively charged adsorbent and anionic PFOA.

**Figure 5:**
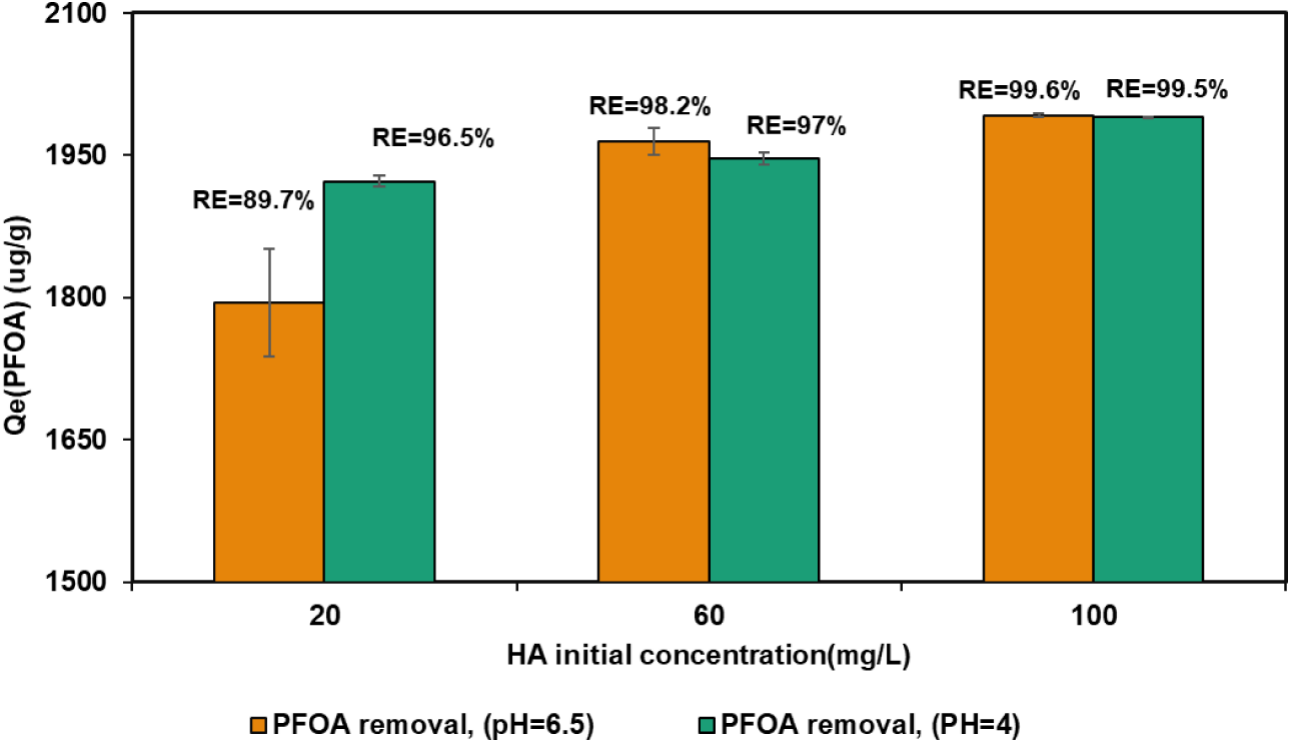
Effect of initial humic acid (HA) concentration (20, 60, and 100 mg/L) on PFOA adsorption by (Cat-CNF) in the presence of HA, at two pH conditions (4.0 and 6.5).

These findings are consistent with the known pH-dependent behavior of PFOA in aqueous solution. At low pH (below its dissociation constant, p*K*_a_ ∼ 2–3), PFOA remains predominantly in the neutral −COOH form, which is poorly soluble and prone to aggregation through hydrogen bonding and hydrophobic clustering of the perfluorinated tails.^52,53^ At higher pH values, PFOA deprotonates to the carboxylate anion (−COO^–^), becoming more soluble and behaving as a typical surfactant and molecules remain dispersed due to electrostatic repulsion, but hydrophobic tail–tail interactions still promote interfacial adsorption and self-assembly.^54,55^ Thus, under acidic conditions, compact HA aggregates combined with neutral or partially protonated PFOA may create favorable domains for coadsorption, explaining the higher removal observed at pH 4 compared to pH 6.5 under low HA concentration.^38,56–59^ As the HA concentration increased to 60 and 100 mg L^–1^, PFOA adsorption improved consistently at both pH values, and the variation between acidic and near-neutral conditions became negligible. This behavior indicates that at higher HA loadings, hydrophobic HA–PFOA associations and multilayer adsorption on the Cat-CNF surface dominate the removal process, thereby minimizing the influence of pH.

These findings collectively indicate that while PFOA sorption is pH-dependent under low HA concentrations, this sensitivity is diminished as HA levels increase. Thus, humic acid not only enhances PFOA uptake but also provides system robustness across a range of pH values. This behavior contrasts with previous studies involving anion exchange resins or GAC, where PFOA removal showed stronger pH dependence and competition from HA at elevated pH.^60,61^ In the present system, the synergistic interplay between cationic CNF, HA, and PFOA reduces the need for pH adjustment and suggests practical application potential under diverse water chemistries.

### 3.5 Adsorption Kinetics and Isotherms of PFOA on the Cat-CNF + HA System

The time-dependent uptake of PFOA was evaluated using the Cat-CNF/HA hybrid adsorbent at an initial PFOA concentration of 2000 μg L^–1^ (Figure 6). The adsorption capacity (Q_e_) increased rapidly within the first few hours, reaching approximately 1.89 mg g^–1^ (corresponding to ∼94.6% removal) at 4 h. After this point, the uptake remained nearly stable throughout the 96 h contact period. This efficient and consistent adsorption behavior can be attributed to the combined influence of humic acid and cationic cellulose nanofibrils. The high HA affinity toward Cat-CNF originates from electrostatic attraction between its anionic carboxyl and phenolic groups and the quaternary ammonium functionalities on the nanofiber surface.

**Figure 6:**
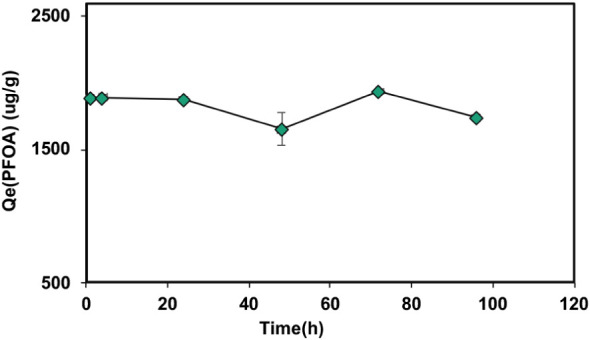
Adsorption kinetics of PFOA by (Cat-CNF + HA) at pH = 4.0 ± 0.2.

Once adsorbed, HA molecules form a uniform coating that not only saturates the cationic sites but also introduces hydrophobic domains. These nonpolar regions facilitate the coadsorption of PFOA, whose fluorinated tails interact with the hydrophobic segments of the HA layer. Consequently, the consistent PFOA adsorption observed over time reflects both direct electrostatic binding and indirect stabilization through HA-assisted hydrophobic association.^36,47,62,63^ Compared with conventional adsorbents such as granular activated carbon (GAC), which typically require 24–48 h to reach equilibrium^64^ due to intraparticle diffusion limitations, the Cat-CNF/HA hybrid exhibited substantially faster adsorption kinetics. Rapid uptake of humic substances and PFAS has similarly been reported for other cationic nanocellulose systems, confirming the efficiency of electrostatically driven, surface-accessible nanofiber adsorbents. Nonlinear fitting to pseudo-first-order and pseudo-second-order models yielded poor correlation (*R* ^2^ ≈ 0), further supporting that PFOA adsorption in the Cat-CNF/HA hybrid is a fast, surface-controlled process, dominated by strong electrostatic and hydrophobic interactions rather than diffusion or chemisorption.^22,36,65^

To further investigate the adsorption mechanism and equilibrium behavior of PFOA on the Cat-CNF/HA hybrid, isotherm models were applied to describe the relationship between equilibrium concentration and adsorption capacity. The resulting uptake increased steeply with increasing concentration, rising from roughly 0.2 mg g^–1^ at the lowest PFOA levels to more than ∼1.9 mg g^–1^ at moderate concentrations, and approached a plateau of ∼0.23 g g^–1^ at the highest loadings. This trend is indicative of site saturation at high coverage and suggests that a finite number of active sites control uptake. The equilibrium data were interpreted using the classic Langmuir (1918) and Freundlich (1926) models^66,67^ (eqs 1 and 2), and the resulting parameters are summarized in Figure 7. The corresponding mathematical expressions are provided below. The Langmuir equation is expressed as

1 $$\mathrm{Langmuir model}\;q_{e}=\frac{k_{L}q_{m}C_{e}}{1+K_{L}C_{e}}$$

**Figure 7:**
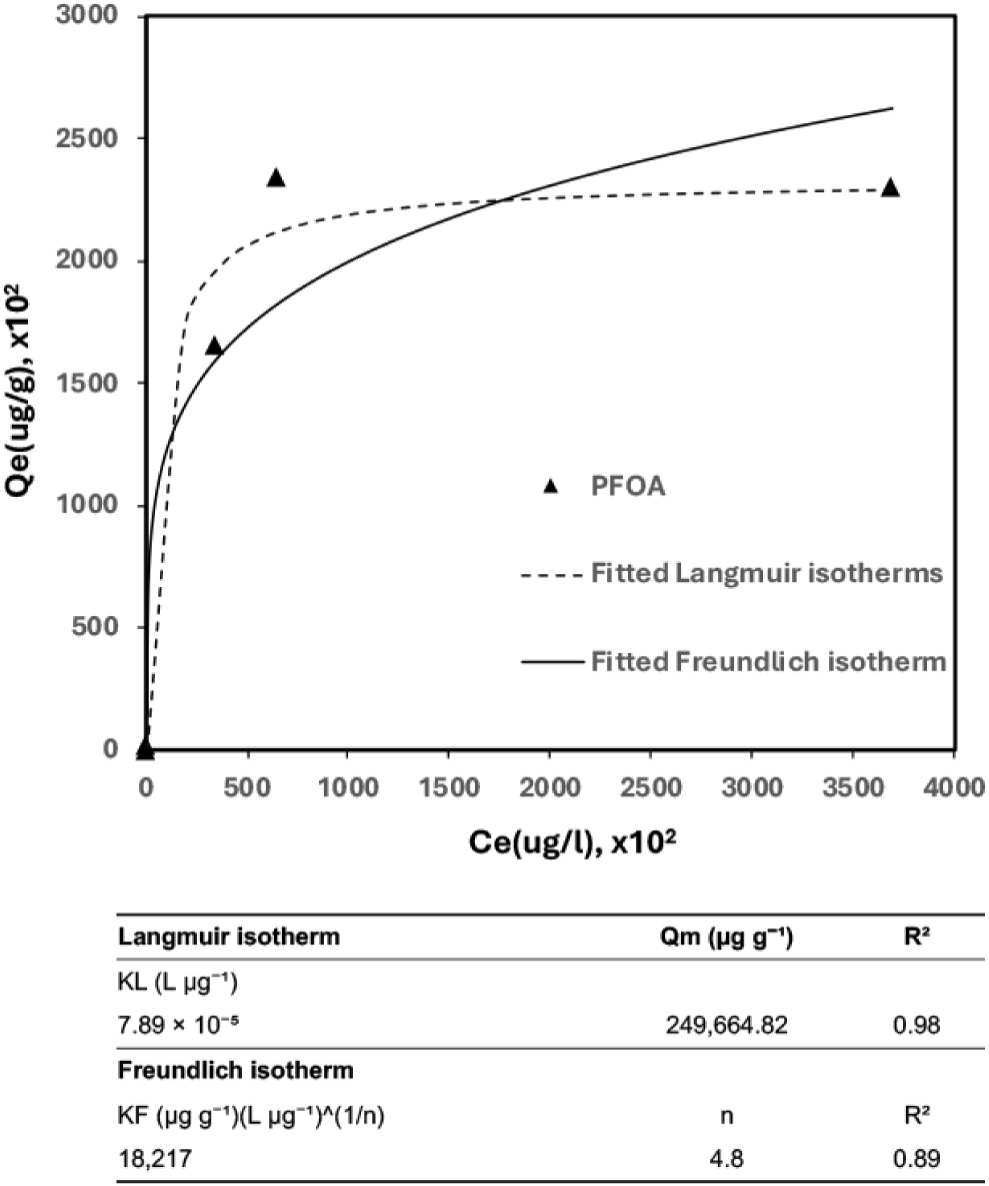
Sorption isotherms of PFOA by (Cat-CNF + HA) at pH = 4.0 ± 0.2.

Where *q*_*e*_ (mg g^–1^) is the equilibrium adsorption capacity, *C_e_* (mg L^–1^) is the equilibrium concentration of PFOA, *q*_*m*_ (mg g^–1^) is the maximum sorption capacity, and *K_L_* (L mg^–1^) is the Langmuir adsorption constant. The Freundlich equation has the form:

2 $$\mathrm{Freundlich model}:qe=K_{F}Ce^{n}$$

Where *K*_*F*_ [(μg g^–1^)­(L μg^–1^)] and *n* is measure of the nonlinearity involved.

The adsorption isotherm of PFOA on the Cat-CNF/HA hybrid was well fitted by both the Langmuir and Freundlich models (Figure 7). The Langmuir fit gave a maximum adsorption capacity (Qm) of 249,664.8 μg g^–1^ and a Langmuir constant (*KK_L_*) of 7.89 × 10^–5^ L μg^–1^, with an *R* ^2^ of 0.98, indicating an excellent fit. The Freundlich model also provided a reasonable description, with *KK_f_* = 18,217, *n* = 4.8, and *R* ^2^ = 0.89, suggesting slightly heterogeneous but favorable adsorption. The isotherm curve exhibited a sharp increase in PFOA uptake at low equilibrium concentrations, followed by a plateau at around 250 mg g^–1^, confirming site saturation and supporting the Langmuir monolayer assumption.

The critical micelle concentration (CMC) of PFOA is reported to be around 25 mM,^47,68^ while the concentrations used in this study ranged from 0.0005 to 1.45 mM, which are below the CMC. This indicates that micelle or hemimicelle formation is unlikely under these conditions, and the observed adsorption is primarily due to direct interactions with the adsorbent surface. Mechanistically, PFOA adsorption is governed by electrostatic attraction between its carboxylate group and the cationic quaternary ammonium groups of Cat-CNF, as well as significant hydrophobic interactions between PFOA’s perfluorinated tail and HA. Humic acid’s nonpolar aromatic domains enhance PFOA uptake through hydrophobic partitioning, promoting HA–PFOA complexation.^69^ Cat-CNF rapidly captures HA and, to a lesser extent, PFOA in mixed systems. Adsorption is primarily driven by electrostatic interactions, while HA enhances PFOA retention through hydrophobic attraction. HA coatings have been shown to immobilize PFOA and increase its retention even on mineral surfaces, supporting this mechanism. These dual electrostatic and hydrophobic interactions explain the high adsorption capacity and good fit to isotherm models. The findings are consistent with previous reports on PFAS binding to positively charged CNF surfaces.^70–72^

### 3.6 Desorption-Regeneration

The regeneration performance of the Cat-CNF and Cat-CNF/HA systems was evaluated through multiple adsorption–desorption cycles to investigate their ability to reversibly bind and release PFOA. Sodium chloride (NaCl) was employed as the regenerant to reduce electrostatic interactions by screening the positive surface charges of Cat-CNF and weakening its attraction to anionic PFOA. This ionic strength effect facilitates desorption while maintaining the structural stability of the adsorbent.^71,73^ In the Cat-CNF system (Figure 8a), the first adsorption cycle achieved high PFOA uptake, with substantial desorption in the following step. However, in the second cycle, both adsorption capacity (Q_e_) and desorption efficiency (DE%) declined significantly. This drop is attributed to residual PFOA from the previous cycle (Q_Remain_ ≈ 720 μg g^–1^) occupying active sites, limiting further adsorption and weakening electrostatic attraction due to site saturation. Interestingly, desorption improved slightly in this round, likely due to displacement of loosely held PFOA by NaCl ions as surface crowding increased.

**Figure 8:**
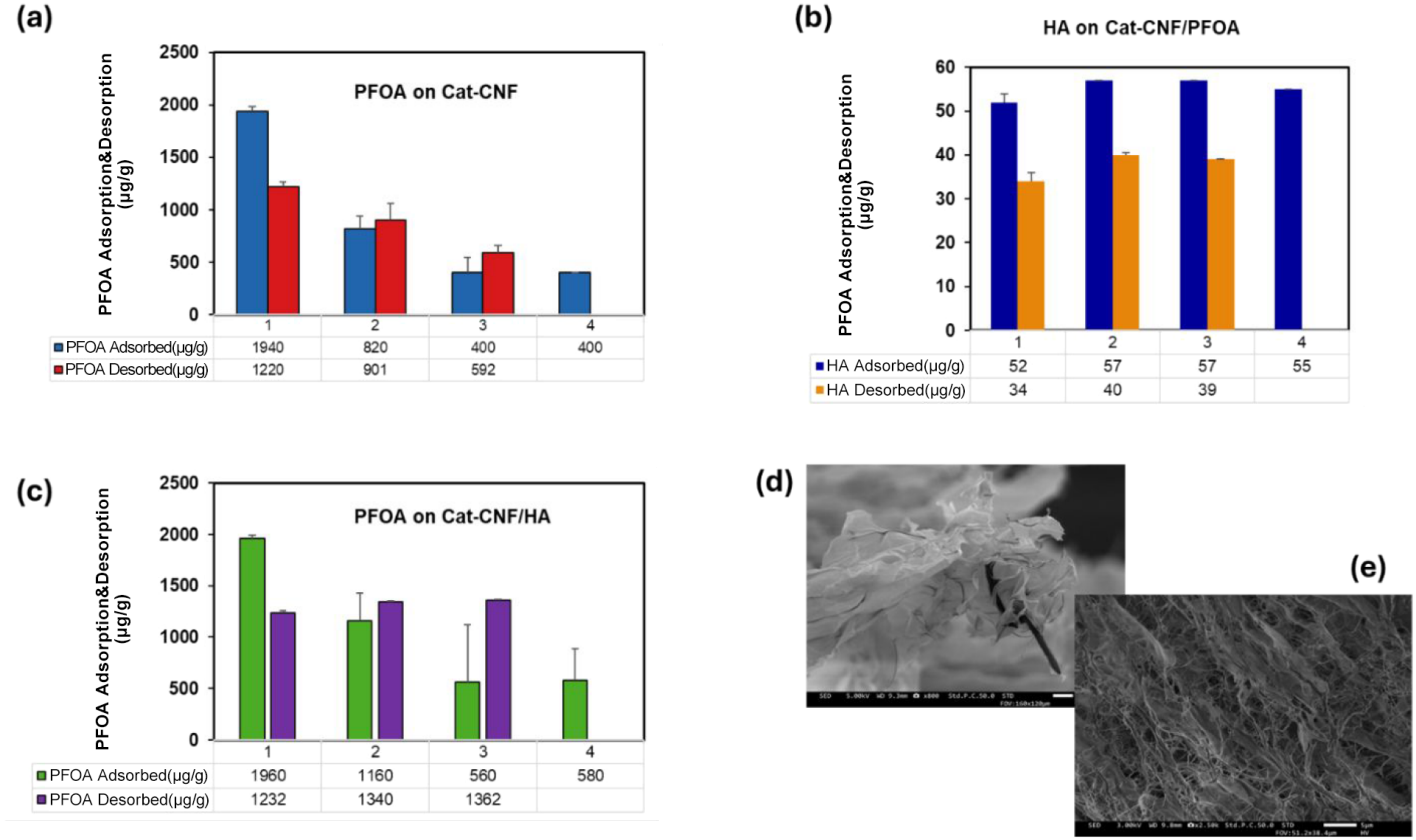
(a) Cyclic regeneration of PFOA on Cat-CNF in NaCl (0.6 M) (b) Cyclic regeneration of HA on Cat-CNF/PFOA in NaCl (0.6 M) (c) Cyclic regeneration of PFOA on Cat-CNF/HA in NaCl (0.6 M) and SEM image of freeze-dried Cat-CNF (d) with adsorbed HA and PFOA (e) after three adsorption–desorption cycles of PFOA.

In the third cycle, Q_e_ remained low, and desorption efficiency decreased again, despite a Q_Remain_ of approximately 639 μg g^–1^. This indicates more irreversible retention of PFOA, as the accumulation of strongly bound residuals from earlier cycles likely hindered further regeneration. These results suggest that while Cat-CNF is initially effective, its regeneration performance diminishes with cycling due to the progressive buildup of persistent PFOA residues.

In contrast, the Cat-CNF/HA hybrid system (Figure 8b and c) demonstrated more dynamic regeneration behavior. In early cycles, HA enhanced adsorption through the formation of hydrophobic domains that promoted PFOA association. Although this led to lower initial desorption efficiency due to strong HA–PFOA interactions, subsequent cycles showed improved PFOA release. The increase in desorption efficiency, particularly in the third cycle, suggests that HA facilitated cumulative saturation of hydrophobic domains, reducing binding strength and enhancing release.

By the second cycle after desorption, Q_Remain_ had increased to around 728 μg g^–1^, and in the third cycle it reached roughly 548 μg g^–1^, suggesting that retained PFOA, while still present, was more readily released in the presence of HA. The presence of HA likely contributes to a more heterogeneous surface environment that enables reversible PFOA association via both electrostatic and hydrophobic mechanisms. While HA remained more strongly retained, its persistent presence on the surface contributed to enhanced desorption of PFOA over time by modulating surface polarity and interfering with tight binding.

These findings indicate that while Cat-CNF loses efficiency with cycling due to residual PFOA accumulation, the hybrid Cat-CNF/HA system maintains robust adsorption and significantly enhanced desorption in later stages. The presence of humic acid supports both improved PFOA retention in early stages and its release in later cycles, likely due to increased surface heterogeneity and hydrophobic interactions.

The SEM images (Figure 8e) show the surface morphology of Cat-CNF after three adsorption–desorption cycles involving humic acid (HA) and PFAS. The low- and high-magnification views confirm that the nanofibrillar network remains largely intact, although signs of surface restructuring are evident compared to the Cat-CNF before regeneration cycles (Figure 8d). In particular, partial collapse of the fibrillar network and the appearance of dense agglomerates suggest the presence of residual adsorbates and possible structural rearrangement caused by repeated NaCl regeneration. Despite these morphological changes, the preservation of the porous matrix highlights the mechanical robustness and reusability of Cat-CNF for water treatment applications.

## 4 Conclusions

This work demonstrates that a cationic cellulose nanofibril–humic acid (Cat-CNF/HA) hybrid matrix provides a synergistic platform for the simultaneous removal of PFOA and humic acid from aqueous solutions. Introducing humic acid creates a stable surface rich in hydrophobic domains, enabling both electrostatic and hydrophobic interactions with PFOA and resulting in rapid, high-efficiency uptake across a wide range of pH and concentrations.

Regeneration experiments revealed that PFOA became progressively easier to desorb over successive cycles while humic acid remained strongly bound, suggesting that the humic coating modulates PFOA binding strength without compromising the structural integrity of the adsorbent. Collectively, these findings establish the Cat-CNF/HA hybrid as a sustainable and effective adsorbent for PFOA removal from high-concentration contaminated water sources such as AFFF-impacted groundwater and industrial effluents. Further studies are needed to evaluate the removal of other PFAS classes with varying chain lengths and headgroup chemistries, including anionic, cationic, and neutral compounds.

## Supplementary Material



## Notes

Zeta potential measurements of cationic cellulose nanofibers (Cat-CNF) at different pH values and in the presence of humic acid; conductometric titration methodology for the determination of surface charge and degree of substitution representative titration curves; SEM and cryo-SEM micrographs of Cat-CNF before and after adsorption experiments; detailed liquid chromatography–mass spectrometry (LC–MS) operating conditions, dilution procedure, LC gradient program, mass spectrometric parameters for PFOA analysis; EDS images of Cat-CNF after PFAS adsorption (PDF)

## Abbreviations

AFM: atomic force microscopy
AGU: anhydroglucose unit
ATR: attenuated total reflectance
Cat-CNF: cationic cellulose nanofiber
C_e_: equilibrium concentration
Cd: equilibrium concentration after desorption
CNF: cellulose nanofiber
DS: degree of substitution
EDS: energy-dispersive X-ray spectroscopy
EPTMAC: (2,3-epoxypropyl)­trimethylammonium chloride
FTIR: Fourier-transform infrared spectroscopy
HA: humic acid
HCl: hydrochloric acid
LC–MS: liquid chromatography–mass spectrometry
MW: molecular weight
NaCl: sodium chloride
NaOH: sodium hydroxide
PFAS: per- and polyfluoroalkyl substances
PFOA: perfluorooctanoic acid
Q_e_: adsorption capacity (mg/g)
Qmax: maximum adsorption capacity (mg/g)
RE: removal efficiency (%)
SD: standard deviation
SEM: scanning electron microscopy
UV–Vis: ultraviolet–visible spectroscopy
ζ-potential: zeta potential
