# Supplementary material for: Concomitant Removal of Perfluorooctanoic Acid (PFOA) and Humic Acid from Water Using a Cationic Cellulose Nanofiber
Source: ACS Omega. 2026 May 19;11(21):31588–99. doi: 10.1021/acsomega.6c02078 (PMC13234679; doi:10.1021/acsomega.6c02078)
Supplement: Supplementary file 1 [file ao6c02078_si_001.pdf]

Supporting Information

Concomitant removal of Perfluorooctanoic Acid (PFOA) and Humic Acid from Water Using a Cationic Cellulose Nanofiber

Azinsadat Jahani Javanmardi[1], Merle Plassmann[2], Aji P. Mathew[1]*

[1].Department of Chemistry, Stockholm University, 106 91 Stockholm, Sweden.

[2]. Department of Environmental Science Stockholm University, 106 91 Stockholm, Sweden.

*Correspondence: aji.mathew@su.se (A.P.M.)

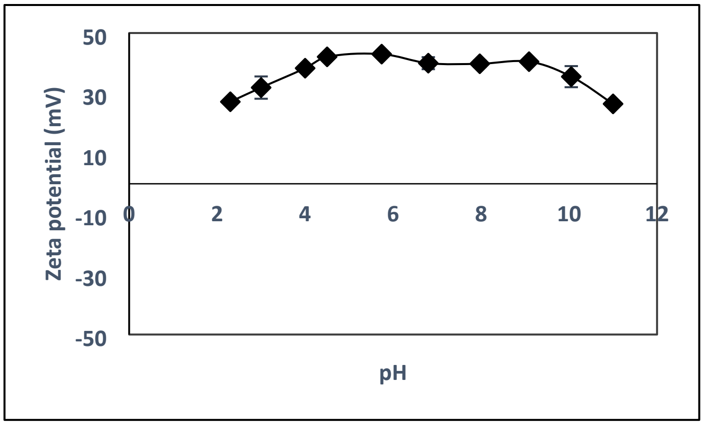

Figure S1. Zeta potential of cationic cellulose nanofibers (Cat-CNF) measured at different pH values.

Conductometric titration

The surface charge and degree of substitution (DS) of the cationic cellulose nanofibril (Cat-CNF) sample were determined using conductometric titration with silver nitrate ($AgNO_3$) solution. The titration was performed using a conductivity meter (SevenExcellence, Mettler Toledo, Switzerland) to monitor the reaction between chloride counterions and Ag^+. It was assumed that each quaternary ammonium group ((CCH3)3N^+) is associated with one chloride ion, allowing the chloride content to be used as a measure of surface charge.

In this procedure, 100 mL of a 0.1 wt% Cat-CNF suspension (containing 0.1 g of dry Cat-CNF) was titrated with 0.1 M $AgNO_3$(aq), with ~0.05 mL increments added every 30 seconds. The content of trimethylammonium groups, expressed in mmol per gram of cellulose (X), was calculated using Equation (1):

$$X=VC/m \qquad \text{(eq.1)}$$

where C is the concentration of $AgNO_3$ (mol L^{-1}), V is the volume of $AgNO_3$ added at the equivalence point (mL), and m is the dry weight of the Cat-CNF sample (g).

The degree of substitution (DS), representing the number of cationic groups per anhydroglucose unit (AGU), was calculated using Equation (2):

$DS=VL .c/(m/162)$ (eq. 2)

where VL is the volume of $AgNO_3$ added (in L), c is the $AgNO_3$ concentration (mol L^{-1}), and 162 g/mol is the molar mass of an AGU[1,2].

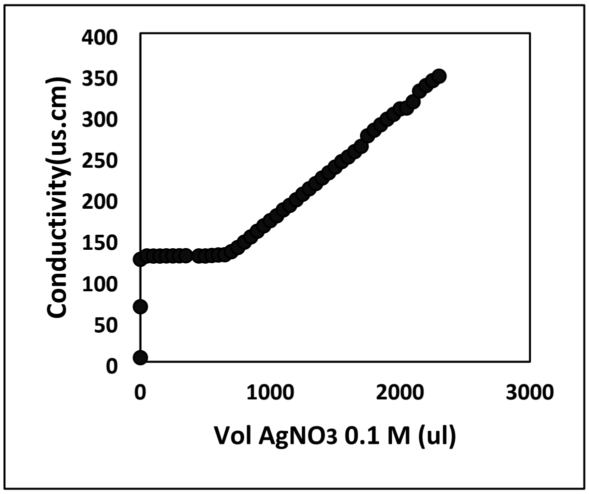

Figure. S2. Example of Conductometric titration curve of Cat-CNF.

SEM micrographs

The Cat-CNFgel samples from adsorption tests (humic acid, PFAS, and co-adsorption systems) were prepared for Cryo-SEM analysis. Small droplets of the sample were initially deposited directly into liquid nitrogen for rapid freezing at -196°C. Once solidified, the frozen samples were carefully mounted onto pre-cooled cryo stubs, ensuring continuous immersion in liquid nitrogen to prevent thawing and to preserve the native distribution of adsorbed contaminants within the fibrillar network. The stub assembly was immediately transferred to the liquid nitrogen bath of the Leica EM VCM system for cryo-fixation, where it was immersed for 3–5 minutes at -196°C. This cryo-fixation process prevented the formation of ice crystals that could distort the delicate nanostructure and facilitated the transfer of the samples under

cryogenic conditions to the coater system (Leica ACE600) using the cryo-vacuum transfer unit (Leica EM VCT500).

During the transfer, the samples were maintained at a temperature of -119°C under vacuum conditions (pressure < 8.0 × 10^{-4} mbar) to prevent condensation and structural collapse. In the coater system, the samples underwent a 24-hour drying process under vacuum to remove residual moisture while preserving their morphology. Subsequently, a 4-nm gold coating was applied to the samples using the coater system to enhance conductivity and improve imaging quality. Finally, high-resolution SEM images were captured by using JSM-IT800 to study the morphology of the cationic cellulose nanofibers before and after the adsorption process[3].

Liquid Chromatography–Mass Spectrometry (LC/MS)

PFAS concentrations were determined using a liquid chromatography–mass spectrometry (LC–MS) system, following the method described in a previously published study[4,5]. The filtrates were diluted as needed to ensure that the final PFAS concentration remained within the instrument's quantifiable range (≤100 µg/L). Different dilution procedures were applied depending on the initial concentration of each sample. For samples at 200 µg/L, 500 µL of filtrate was mixed with 500 µL of LC-MS grade methanol. Samples at 2000 µg/L were prepared by combining 50 µL of filtrate with 500 µL of methanol and 450 µL of ultrapure water. For highly concentrated samples (200 mg/L), 500 µL of filtrate was mixed with 500 µL of methanol and 500 µL of water, followed by a 1000-fold dilution. All prepared solutions were transferred to LC-MS vials for analysis. Instrumental analysis was carried out using an ultra-high-performance liquid chromatography (UHPLC) system connected to a Quantiva triple quadrupole mass spectrometer (Thermo Fisher Scientific), operating in negative electrospray ionization (ESI−) mode. A BEH C18 column (50 × 2.1 mm, 1.7 µm particle size, Waters) was used for separation.

The mobile phase consisted of solvent A (95% water, 5% acetonitrile, 2 mM ammonium acetate) and solvent B (95% acetonitrile, 5% water, 2 mM ammonium acetate), applied through a 10-minute gradient program. The flow rate was kept constant at 0.4 mL/min. Detailed LC gradient conditions are provided in Table 1.

TableS1. Detailed LC gradient conditions.

Time (min)	Mobile Phase A (%)	Mobile Phase B (%)	Flow Rate (mL/min)
0	90	10	0.4
0.5	90	10	0.4
5	20	80	0.4
5.1	0	100	0.4
8	0	100	0.4
10	90	10	0.4

Mass spectrometric detection of PFOA was performed in multiple reaction monitoring (MRM) mode, using a quantifier transition of m/z 413 → 369 and a qualifier transition of m/z 413 → 169. The mass spectrometer was operated with the following source parameters: the ion transfer tube was set to 350 °C, the vaporizer to 450 °C, and the sheath and auxiliary gas flows to 40 and 15 arbitrary units, respectively. A spray voltage of 2.5 kV was applied. These conditions are summarized in Table 2. Quantification was based on the comparison of peak areas among sample extracts.

TableS2. Mass spectrometric detection conditions

Parameter	Value
Ion transfer tube temp	350 °C
Vaporizer temp	450 °C
Sheath gas (au)	40
Auxiliary gas (au)	15
Spray voltage	2.5 kV

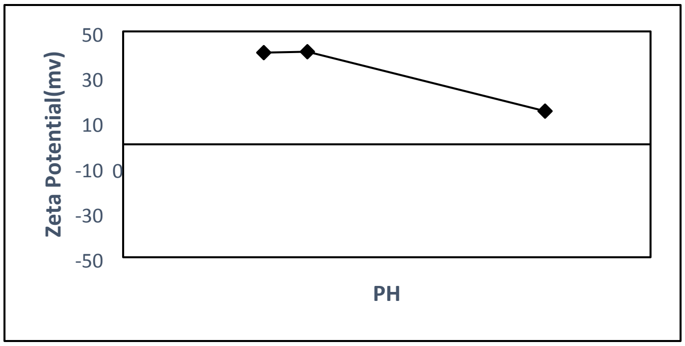

Figure S3. Zeta potential of Cat-CNF in the presence of 60 mg/L humic acid at varying pH.

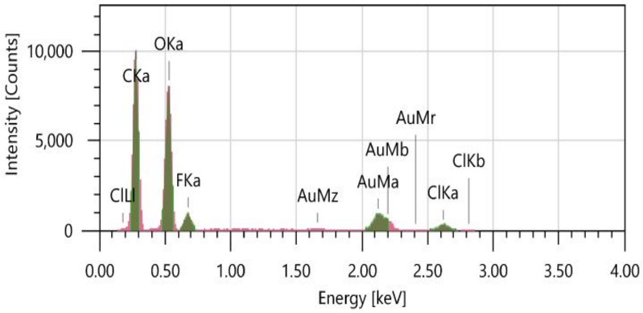

Figure S4. EDS images of Cat-CNF after PFAS adsorption.

References

(1) (PDF) Cationic Surface Functionalisation of Cellulose Nanocrystals. *ResearchGate*. https://doi.org/10.1039/b806789a.

(2) Bianchi, S.; França, D.; Faez, R. Anionic and Cationic Cellulose Nanofibrils as a Macronutrient-Carrying Vehicle. *Cellulose* **2024**, *31* (2), 1053–1070. https://doi.org/10.1007/s10570-023-05662-2.

(3) *Cryo-SEM Investigation of Chlorella Using Filter Paper as Substrate*. https://bio-protocol.org/en/bpdetail?id=5143&type=0 (accessed 2025-05-13).

(4) Schultes, L.; Vestergren, R.; Volkova, K.; Westberg, E.; Jacobson, T.; P. Benskin, J. Per- and Polyfluoroalkyl Substances and Fluorine Mass Balance in Cosmetic Products from the Swedish Market: Implications for Environmental Emissions and Human Exposure. *Environ. Sci. Process. Impacts* **2018**, *20* (12), 1680–1690. https://doi.org/10.1039/C8EM00368H.

(5) Spaan, K. M.; van Noordenburg, C.; Plassmann, M. M.; Schultes, L.; Shaw, S.; Berger, M.; Heide-Jørgensen, M. P.; Rosing-Asvid, A.; Granquist, S. M.; Dietz, R.; Sonne, C.; Rigét, F.; Roos, A.; Benskin, J. P. Fluorine Mass Balance and Suspect Screening in Marine Mammals from the Northern Hemisphere. *Environ. Sci. Technol.* **2020**, *54* (7), 4046–4058. https://doi.org/10.1021/acs.est.9b06773.
